# An Enhanced SMS Text Message–Based Support and Reminder Program for Young Adults With Type 2 Diabetes (TEXT2U): Randomized Controlled Trial

**DOI:** 10.2196/27263

**Published:** 2021-10-21

**Authors:** Timothy Middleton, Maria Constantino, Margaret McGill, Mario D'Souza, Stephen M Twigg, Ted Wu, Aravinda Thiagalingam, Clara Chow, Jencia Wong

**Affiliations:** 1 Diabetes Centre Royal Prince Alfred Hospital Camperdown Australia; 2 Royal Prince Alfred Clinic Charles Perkins Centre The University of Sydney Camperdown Australia; 3 Faculty of Medicine and Health University of Sydney Camperdown Australia; 4 Sydney Local Health District Clinical Research Centre Camperdown Australia; 5 Department of Cardiology Westmead Hospital Westmead Australia; 6 Westmead Applied Research Centre The University of Sydney Westmead Australia; 7 Cardiovascular Division The George Institute for Global Health Sydney Australia

**Keywords:** young-onset type 2 diabetes, SMS, clinic attendance, engagement, diabetes, digital health, mobile health, adolescents

## Abstract

**Background:**

Clinic attendance, metabolic control, engagement in self-management, and psychological health are suboptimal in young-onset (age of onset <40 years) type 2 diabetes.

**Objective:**

We examined the effectiveness of an enhanced SMS text message–based support and reminder program in improving clinic attendance, metabolic control, engagement in self-management, and psychological health in young-onset type 2 diabetes.

**Methods:**

A 12-month, parallel-arm, randomized controlled trial comparing an enhanced, semipersonalized SMS text message–based intervention (incorporating 1-8 supportive and/or informative text messages per month) against standard care was conducted in a specialized clinic for young adult type 2 diabetes. The primary outcome was maintenance of 100% attendance at scheduled quarterly clinical appointments. Secondary outcomes included (1) metabolic indices, (2) pathology and self-monitored blood glucose (SMBG) data availability, and (3) psychosocial well-being.

**Results:**

A total of 40 participants were randomized, and 32 completed their 12-month study visit. The average participant age was 32.7 (SD 5.1) years, 50% (20/40) were male, and baseline glycated hemoglobin A_1c_ (HbA_1c_) was 7.3% (SD 1.9%) (56 mmol/mol, SD 20). A higher proportion of the intervention group achieved 100% attendance (12/21, 57%, vs 5/19, 26%, for the control group); Kaplan-Meier analysis demonstrated significantly greater cumulative attendance in the intervention group (*P*=.04). There were no between-group differences in HbA_1c_, BMI, lipids, or availability of pathology and SMBG data. Odds of recording an improvement in the Diabetes Empowerment Scale–Short Form score were higher in the intervention group at 6 months (odds ratio [OR] 4.3, 95% CI 1.1-17), with attenuation of this effect at study end (OR 3.1, 95% CI 0.9-11). Program acceptability was high; >90% of participants would recommend the program to new patients.

**Conclusions:**

An enhanced SMS text message–based support and reminder program doubled scheduled clinic attendance rates for patients with young-onset type 2 diabetes. The program was highly acceptable and provided early support for patient empowerment but had no significant effect on measures of metabolic control or self-management.

**Trial Registration:**

Australian and New Zealand Clinical Trials Registry (ACTRN12618000479202); https://www.anzctr.org.au/Trial/Registration/TrialReview.aspx?id=373579

## Introduction

Over the past 20 years, young‑onset type 2 diabetes (age of onset <40 years) has emerged as a significant clinical problem. The worldwide prevalence of young‑onset type 2 diabetes more than doubled during the decade spanning 2003 to 2013, and today, well in excess of 60 million young people are living with type 2 diabetes [1,2]. This is particularly concerning given the aggressive nature of young-onset type 2 diabetes, with high rates of complications, established premature mortality, and the poorest comparative outcomes [3-6].

Suboptimal clinic attendance and poor engagement in self-management represent significant barriers to improving long-term outcomes [7,8]. There is specific evidence that clinic attendance associates with favorable outcomes in young-onset type 2 diabetes; during the landmark Treatment Options for Type 2 Diabetes in Adolescents and Youth (TODAY) study of adolescent type 2 diabetes, it was established that participants who attended ≥75% of scheduled lifestyle education sessions achieved significantly greater weight reductions than those who attended less often [9]. An audit of clinic attendance at a metropolitan, hospital-based diabetes center clearly demonstrated that young adults with type 2 diabetes were the subgroup that had the highest rate of nonattendance; median loss to follow-up time was approximately 4 months [10]. As each clinical encounter provides an excellent opportunity to review a patient’s understanding of diabetes and its management, convey important messages relating to self-management, adjust pharmacotherapy, perform complications screening, and foster a therapeutic rapport, novel ideas are needed to motivate young adults with type 2 diabetes and encourage more reliable clinic attendance.

In recent years, innovative text messaging interventions have yielded promising results in high-risk groups. The landmark TEXTME study demonstrated that a structured, lifestyle-based SMS text messaging program could improve cardiovascular risk factor management after myocardial infarction [11]. In adolescent medicine, SMS text message interventions have been used to address important issues, including teenage pregnancy and binge drinking of alcohol [12,13]. In the field of diabetes, the SMS4BG study established that an SMS text messaging program could facilitate modest improvement in glycemic control [14]. However, the SMS4BG study did not specifically examine the impact of the program on clinic attendance, nor did it specifically target individuals with young-onset type 2 diabetes. A study from Hong Kong did identify a benefit of an SMS text message reminder for the attendance rate within a type 2 diabetes population at a primary care clinic [15]. However, participants in the Hong Kong study had a mean age of 63 years; as a result, the benefit of SMS text message reminders in the young-onset type 2 diabetes setting remains to be established. Overall, young adults with type 2 diabetes have been underrepresented in most clinical studies to date [16]. Consequently, high-quality evidence to guide management of young-onset type 2 diabetes remains scarce.

It is widely understood that young people are frequent users of information and communications technology. In 2012 and 2017, young adult respondents to our diabetes and technology survey reported the highest rates of mobile phone ownership and a high degree of comfort with the use of technology in diabetes management [17]. Familiarity with SMS text messaging technology as well as its low cost and ready scalability provide a great impetus for an exploration of the utility of SMS text message interventions in the young adult demographic. We hypothesized that an enhanced SMS text message–based support and reminder program would improve clinic attendance, metabolic health, and engagement in diabetes self-management and psychosocial well-being within our young-onset type 2 diabetes cohort. We set out to test this hypothesis in the TEXT2U randomized controlled trial (RCT).

## Methods

### Study Design

TEXT2U was a parallel group, investigator-blinded, 12-month RCT involving participants with young‑onset type 2 diabetes (age of onset 18-40 years). The primary aim was to assess the effect of an enhanced SMS text message–based support and reminder program on clinic attendance. Secondary aims were to assess (1) metabolic outcomes, (2) diabetes self-management practices as evidenced by availability of self-monitoring of blood glucose (SMBG) data and pathology results, and (3) the psychosocial impact of the intervention as measured by change in validated diabetes-specific questionnaire scores (Problem Areas in Diabetes 5-item Short Form (PAID-5) [18], Diabetes Empowerment Scale–Short Form (DES-SF) [19], and Type 2 Diabetes Stigma Assessment Scale (DSAS-2) [20]). The study was approved by the Sydney Local Health District Ethics Review Committee and registered with the Australian and New Zealand Clinical Trials Registry (ACTRN12618000479202).

### Study Setting and Participant Recruitment

The Royal Prince Alfred Hospital (RPAH) Diabetes Centre is a secondary and tertiary referral center that services an ethnically diverse population in metropolitan Sydney, Australia. Individuals were eligible to enter the study if they had an established type 2 diabetes diagnosis, had access to a mobile phone, and were aged 18-40 years at time of enrolment. Individuals with insufficient proficiency in English to read the text messages in the study were excluded. All participants provided written informed consent prior to study entry. Recruitment was performed between March and August 2018.

### Randomization

Block randomization was employed with a standard block size of 4 using a customized, computer-based program. Participants were allocated 1:1 to either the standard care control group or the standard care plus enhanced SMS text message–based support and reminder intervention group. Medical staff delivering patient care were blinded to study group allocation.

### Interventions

#### Control: Clinic Standard of Care

The control group received our young-onset type 2 diabetes clinic standard of care, which is aligned with and realizes current recommendations for youth and young adults with type 2 diabetes [21]. It includes quarterly follow-up appointments with a diabetes educator and endocrinologist. Glycemic control was assessed via glycated hemoglobin A_1c_ (HbA_1c_) and capillary-based SMBG results, weight and blood pressure (BP) were measured, and treatment adjustments were made at the discretion of the treating endocrinologist. Dietary review with an accredited dietitian was provided as required. All participants were given a personal blood glucose meter with a starter pack of glucose monitoring strips and a personal lancing device for SMBG. All individuals in the young adult type 2 diabetes clinic are enrolled in the Australian National Diabetes Services Scheme, which provides ongoing access to subsidized glucose testing strips and lancets.

After each clinic visit, participants were provided with details of their next follow-up appointment. A generic SMS text message reminder was sent prior to scheduled appointments via the hospital’s outpatient management system. In the event of a missed clinic appointment, clinic staff made attempts to contact the relevant individual (via telephone and email) to reschedule a makeup appointment.

#### Intervention: Enhanced SMS Text Message–Based Support and Reminder Program

The enhanced SMS text message–based support and reminder program involved receipt of clinic standard of care as outlined above, plus a structured program of semipersonalized text messages. Message content was individualized on the basis of key baseline characteristics, including gender and smoking status. Message delivery was managed by an automated system developed and programmed by coauthor AT (TextQStream, Python 3.6, using the Pycap 1.02 library). This system was developed previously [22], and customization was performed in-house for the TEXT2U trial. Computer software was run through the University of Sydney Research Electronic Data Capture (REDCap) system [23]. Messages to the study participants were sent through a gateway interface over Australian telephone networks at no cost to individual participants. All SMS text messages sent during the study were logged by the system; the study log included the date and time that each message was delivered. All participants received an introductory message in the week following their baseline visit and then two messages per week for the first 2 months of the study. Text message frequency decreased to one message per week during the third month and then one text message per month thereafter. All study messages were sent at random times during standard business hours: Monday to Friday, 9 AM to 5 PM. Instructions on how to opt out of the messaging program were provided.

The SMS text messaging program was developed by a focus group of 10 diabetes specialists associated with the Royal Prince Alfred Hospital Diabetes Centre; adjustments were made following consumer review by young adults with diabetes. In particular, 15 young adults with diabetes (aged 18-40 years), who did not form part of the young adult type 2 diabetes clinic, were asked to complete a brief survey and each provide feedback on different subsets of messages that were to be featured in the study. Program messages were designed to contain a mix of supportive and informative content. Messages were entirely text-based; emojis and multimedia messaging were not incorporated into the program. A personalized appointment reminder was sent to participants in the enhanced SMS group in the week before each follow-up appointment. Examples of program text messages are included in Multimedia Appendix 1 (Table S1).

An option was provided for enhanced SMS group participants to engage with the study team via the study-specific SMS portal. Questions relating to diabetes and its management could be sent directly to the study team at a participant’s convenience. The SMS portal was actively monitored by a research assistant, and all text messages received from participants were reviewed and replied to within 1 business day.

### Trial Procedures

All participants underwent baseline assessment and were asked to attend quarterly follow-up visits for 12 months. Weight, BP, SMBG records, and routine pathology test results were assessed at each study visit. At baseline, all participants completed the Health Literacy Questionnaire [24]. Validated measures of psychosocial status, including the PAID-5 [18], DES-SF [19], and DSAS-2 [20], were self-completed by participants at baseline, 6 months, and 12 months. Participants assigned to the enhanced SMS intervention group were invited to complete an evaluation of the program via a standardized, study-specific questionnaire at the end of the study.

### Statistical Analysis

From prior analyses [10], the median time to clinic dropout associated with our standard care approach in the young-onset type 2 diabetes clinic was 4 months. Previous studies comparing attendance rates in SMS text message reminder vs control interventions have demonstrated odds ratios (ORs) of 1.7-4.3 in favor of text messaging [25]. Given this information, we estimated that an enhanced SMS text message–based support and reminder system would increase the median time to clinic dropout in our young-onset type 2 diabetes cohort from 4 to 12 months. We determined that a 12-month study with quarterly clinic follow-up for 40 individuals (randomized 1:1 to the intervention and control groups) would have 80% power (α=.05) to enable us to reject the null hypothesis of no difference in loss to follow-up.

The primary outcome was evaluated using the principles of time to event analysis. For secondary outcomes, data collected at each follow-up visit (regardless of whether the visit was attended as scheduled or as a make-up appointment) were considered in the analyses. For those participants who failed to attend a follow-up appointment and did not attend a makeup appointment, missing data were not imputed. Participants were analyzed by original group assignment. Data from descriptive analyses are reported as mean (SD) or n (%) values. Comparisons between groups were made using analysis of variance for continuous variables and Pearson chi-squared tests for categorical variables.

The primary outcome of attendance at all scheduled follow-up appointments was analyzed using a Kaplan-Meier approach. Difference in scheduled follow-up attendance between the intervention and control groups was compared using the log-rank test. In addition to our primary attendance outcome, overall attendance (ie, attendance at scheduled appointments and rescheduled makeup appointments) was assessed.

With respect to secondary outcomes, metabolic indices at 12 months in the control and intervention groups were compared using the independent samples *t* test. Mean changes (from baseline) for metabolic indices in both the control and intervention groups were evaluated using the paired samples *t* test. Binary logistic regression analyses were performed to explore differences between the intervention and control groups in the log odds of observing the other secondary outcomes of interest. For the PAID-5, DES-SF, and DSAS-2 analyses, the outcome of interest was positive change in questionnaire score (ie, a score change that would indicate less diabetes distress and stigma or greater self-efficacy). The dependent variable for each model was the outcome of interest, and the independent variables included group assignment, follow-up time, and the interaction between group assignment and follow-up time. A general estimating equations framework was employed to allow within-participant correlations (in the context of repeated measures) to be taken into account. Data collected from participants who attended make-up appointments were considered in secondary outcome analyses. These data were ascribed to the nearest scheduled follow-up appointment.

Statistical analyses were performed using SPSS, version 24.0 (IBM Corporation). All statistical tests were two-tailed and were conducted at the .05 significance level.

## Results

Between March and August 2018, 41 patients of the RPAH Diabetes Centre young-onset type 2 diabetes clinic were approached regarding study participation; 40 were enrolled and randomized, and 32 participants completed a 12-month follow-up appointment (Multimedia Appendix 2, Figure S1). The mean age of the study cohort was 32.7 years, 50% (20/40) of participants were male, mean HbA_1c_ was 7.3% (56 mmol/mol) and mean duration of diabetes at the time of study entry was 6.4 years. Baseline characteristics were similar in the enhanced SMS and standard of care groups (Table 1). Notably, there were no between-group differences in glycemic control, diabetic pharmacotherapy use, or health literacy measures.

**Table 1:** Baseline characteristics of the study cohort (N=40).

Characteristic		Values		*P* value
		Intervention group (n=21)	Control group (n=19)	
Male gender, n (%)		10 (48)	10 (53)	.99
Age (years), mean (SD)		33.0 (5.8)	32.4 (4.4)	.71
**Ethnic background, n (%)**				.38
	East/Southeast Asian	8 (38)	3 (16)	
	Subcontinental	5 (24)	7 (37)	
	European	4 (19)	6 (32)	
	Other	4 (19)	3 (16)	
Nonsmoker, n (%)		11 (52)	14 (74)	.17
**Education, n (%)**				.14
	TAFE^a^/university	19 (91)	13 (68)	
	High school	2 (10)	5 (26)	
	Not disclosed	0 (0)	1 (5)	
Duration of diabetes (years), mean (SD)		7.6 (6.2)	5.0 (5.9)	.18
**Diabetes treatment, n (%)**				.32
	Diet alone	2 (10)	5 (26)	
	Oral hypoglycemic medication	14 (67)	8 (42)	
	Insulin oral hypoglycemic agent	5 (24)	6 (32)	
HbA_1c_^b^ (%), mean (SD)		7.2 (1.6)	7.3 (2.1)	
**HbA_1c_ range (%), n (%)**				.94
	<6.5	9 (43)	9 (47)	
	6.5-8.5	8 (38)	8 (42)	
	>8.5	4 (19)	2 (10)	
BMI (kg/m^2^), mean (SD)		31.8 (8.6)	31.6 (5.1)	.92
Systolic blood pressure (mm Hg), mean (SD)		118 (11)	118 (9)	.94
Antihypertensive medication, n (%)		7 (33)	4 (21)	.39
Total cholesterol (mmol/L), mean (SD)		4.6 (1.2)	4.7 (0.9)	.87
Triglycerides (mmol/L), mean (SD)		2.1 (1.6)	2.7 (2.1)	.32
Lipid-lowering medication, n (%)		6 (29)	7 (37)	.58
eGFR^c^ (mL/min/1.73 m^2^), mean (SD)		111 (19)	112 (13)	.88
Abnormal UACR^d^, n (%)		8 (38)	6 (32)	.67
HLQ^e^ Scale 2 score^f^, mean (SD)		3.1 (0.3)	3.3 (0.5)	.14
HLQ Scale 6 score^g^, mean (SD)		4.0 (0.7)	4.1 (0.6)	.40

^a^TAFE: Technical and Further Education.

^b^HbA_1c_: glycated hemoglobin A_1c_.

^c^eGFR: estimated glomerular filtration rate.

^d^UACR: urine albumin to creatinine ratio.

^e^HLQ: Health Literacy Questionnaire.

^f^HLQ Scale 2 score reflects an individual’s confidence in their knowledge to manage their health. Higher scores equate to higher confidence levels.

^g^HLQ Scale 6 score reflects an individual’s confidence in their ability to actively engage with health care providers. Higher scores equate to higher confidence levels.

### Primary Endpoint: 100% Attendance at Scheduled Follow-up Appointments

**Figure 1:**
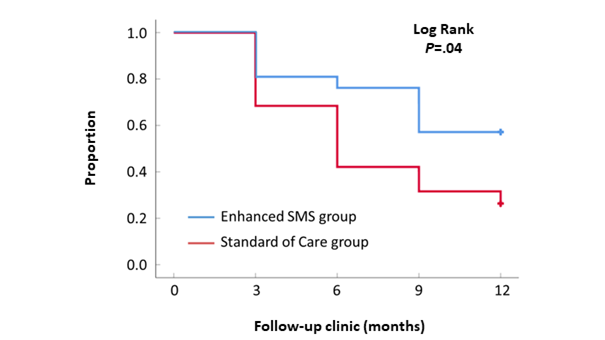
Kaplan-Meier curve illustrating the proportion of participants who maintained 100% attendance as scheduled throughout the study.

The proportion of participants who maintained 100% attendance at scheduled clinic appointments for successive follow-up appointments is presented in Figure 1. At 12 months, more participants in the enhanced SMS intervention group achieved 100% attendance at scheduled clinic appointments (12/21, 57%, vs 5/19, 26%). A statistically significant difference between the intervention and control groups was observed with respect to 100% attendance (Figure 1, Table 2; log-rank *P*=.04).

**Table 2:** Participants in the enhanced SMS and control groups who maintained 100% attendance in the TEXT2U study (N=40).

Group	Value, n (%)				
	Baseline	3 months	6 months	9 months	12 months
Intervention (n=21)	21 (100)	17 (81)	16 (76)	12 (57)	12 (57)
Control (n=19)	19 (100)	13 (68)	8 (42)	6 (32)	5 (26)

### Secondary Endpoints

#### Overall Clinic Attendance

The proportion of clinic appointments that were attended as scheduled was greater in the intervention group than in the control group (64/84, 76%, vs 44/76, 58%; Multimedia Appendix 3, Table S2). It was possible to boost clinic attendance to 80% in both the intervention and control groups, but only after proactive contact by administrative staff and rescheduling of missed appointments (Multimedia Appendix 3, Table S2). The requirement for this extra administrative burden was lower in the intervention group; 18% fewer rescheduling interventions were necessary in this group.

#### Clinical Metabolic Outcomes

Key end-of-study metabolic indices for the intervention and control groups are presented in Table 3. For those with 12-month follow-up data (20/21 intervention and 15/19 control, respectively), there was no difference in mean HbA_1c_ between groups (mean 7.1%, SD 1.1%, for the intervention group vs mean 6.6%, SD 1.7%, for the control group; *P*=.37). For both groups, clinic attendance was associated with glycemic stability; there was no statistically significant change in mean 12-month HbA_1c_ (relative to baseline) for either the intervention or control group (Multimedia Appendix 4, Table S3). If the analysis was restricted to those with 100% attendance, the change in HbA_1c_ from baseline to study end was –0.58% (95% CI –1.70 to 0.59) (intervention) and –0.18% (95% CI –0.46 to 0.10) (control). Although the physicians working in the young adult type 2 diabetes clinic ensure that all patients have access to valid prescriptions for their diabetic pharmacotherapy at each clinic visit, absolute adherence to pharmacotherapy was not actively assessed during this study. Analogously to HbA_1c_, there were no statistically significant between-group differences with respect to BMI, total cholesterol, or triglycerides.

**Table 3:** Mean (SD) values of selected metabolic indices after 12 months for the intervention and control groups.

Metabolic index	Values, mean (SD)			*P* value
	Intervention group	Control group	Mean difference (95% CI)	
HbA_1c_^a^ (%)	7.1 (1.1)	6.6 (1.7)	0.4 (–0.5 to +1.4)	.37
BMI^b^ (kg/m^2^)	30.4 (8.4)	31.8 (5.8)	–1.5 (–6.7 to +3.8)	.57
Total cholesterol^c^ (mmol/L)	4.6 (1.4)	4.7 (0.9)	–0.1 (–1.0 to +0.7)	.75
Triglycerides^c^ (mmol/L)	2.1 (1.7)	2.9 (4.1)	–0.8 (–3.3 to +1.6)	.50

^a^HbA_1c_: glycated hemoglobin A_1c_. 12-month HbA_1c_ data were available for 20/21 intervention group participants and 15/19 control group participants.

^b^12-month BMI data were available for 18/21 intervention group participants and 15/19 control group participants.

^c^12-month total cholesterol and triglyceride data were available for 20/21 intervention group participants and 14/19 control group participants.

#### Diabetes Self-management Practices

**Figure 2:**
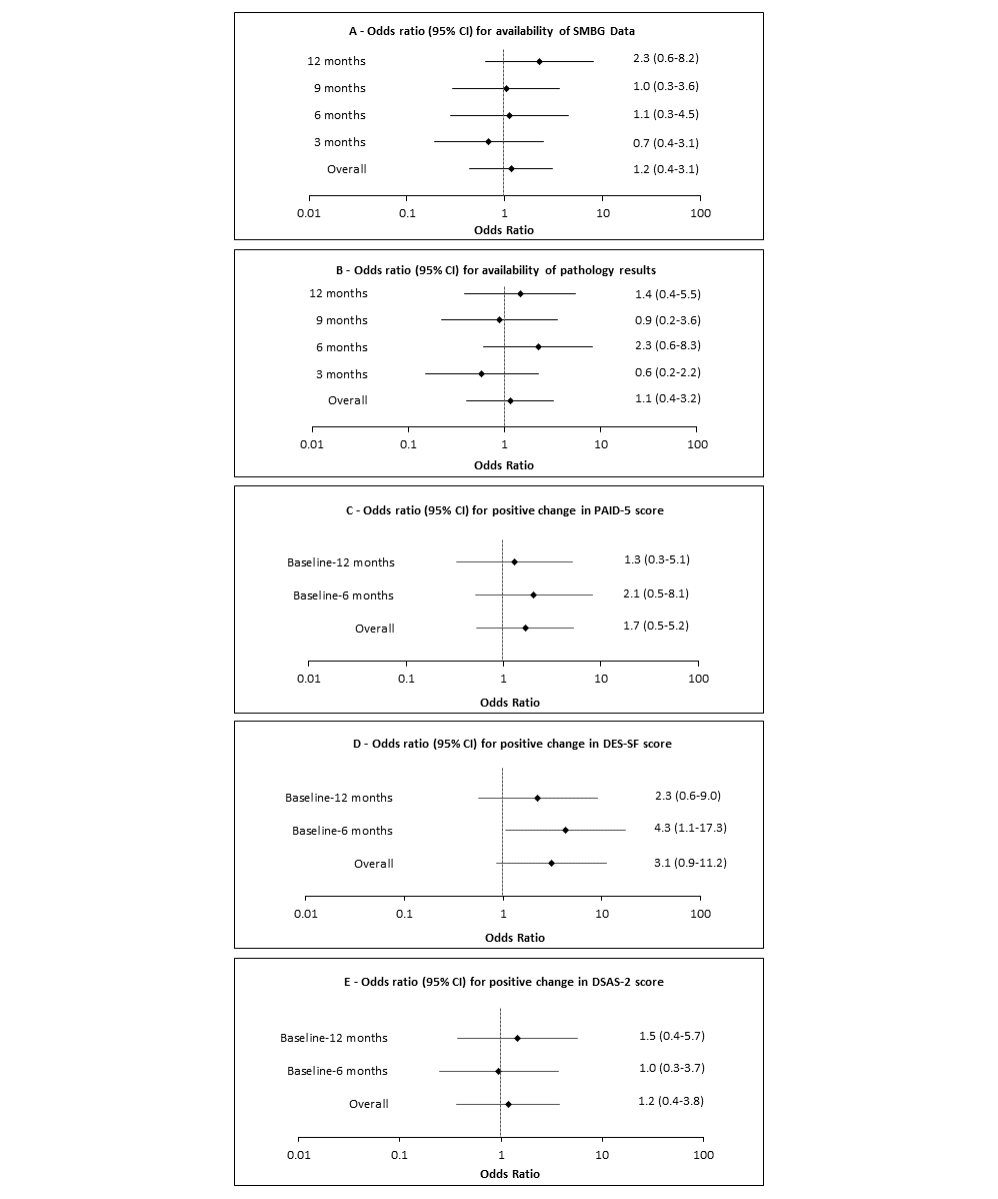
Forest plots illustrating the odds ratios and 95% confidence intervals for positive changes in selected secondary outcomes (A, SMBG; B, pathology results; C, PAID-5 score; D, DES-SF score; E, DSAS-2 score) for the TEXT2U intervention. DES-SF: Diabetes Empowerment Scale-Short Form; DSAS-2: Type 2 Diabetes Stigma Assessment Scale; PAID-5: Problem Areas in Diabetes 5-item Short Form; SMBG: self-monitored blood glucose.

Overall, SMBG data were available at 40% of the intervention group’s appointments, versus 37% of the control group’s appointments (Multimedia Appendix 5, Figure S2). The corresponding figures for the pathology results were 69% and 66%, respectively (Multimedia Appendix 6, Figure S3). Binary logistic regression modeling revealed no between-group differences with respect to availability of SMBG or pathology results at follow‑up. The ORs for availability of SMBG data and pathology results in the intervention group were 1.2 (95% CI 0.4-3.1) and 1.1 (95% CI 0.4-3.2), respectively (Figure 2, Panels A and B).

### Psychosocial Impact (Baseline Versus 6 Months and 12 Months)

An overview of the PAID-5, DES-SF, and DSAS-2 scores for the study cohort is presented in Multimedia Appendix 7 (Table S4). Analysis of the changes in DES‑SF score at 6 months revealed a significant between-group difference favoring the intervention (Multimedia Appendix 7, Table S4); 68% (13/19) of members of the intervention group recorded a favorable change in DES-SF score at 6 months, versus 31% (5/16) in the control group (*P*=.03). At 12 months, this difference was no longer statistically significant. In the analyses of the changes in PAID-5 and DSAS-2 scores, there were no significant between-group differences for either of the scores at 6 or 12 months (Multimedia Appendix 7, Table S4).

Binary logistic regression modeling revealed no evidence of a difference in the odds of recording a positive change in PAID‑5 or DSAS-2 scores for the intervention and control groups (Figure 2, panels C and E). Although not statistically significant at 12 months, the OR for positive change in DES-SF score at 6 months (4.3, 95% CI 1.1‑17.3) was significantly higher for those who participated in the enhanced SMS text messaging program (Figure 2, panel D).

### Evaluation of the Enhanced SMS Text Message–Based Support and Reminder Program

Of the 21 participants randomized to the enhanced SMS text messaging intervention group, 20 received all study text messages; 1 participant asked to stop receiving text messages midstudy. Of the 21 participants randomized to the enhanced SMS group, 11 chose to use the study-specific SMS portal (Multimedia Appendix 8, Table S5). The median number of messages sent by participants was 3 (range 1-29). Analysis of message content revealed that 28% (21/74) of the messages sent by participants expressed gratitude (for a message received or a service provided by the clinic), 25% (19/74) of messages directed a diabetes-specific question to the study team or sought clarification regarding some aspect of diabetes management, and 24% (18/74) of messages were concerned with rescheduling a clinic appointment. With respect to diabetes-related questions, participants inquired about a variety of different issues, including management of hypoglycemia, dietary recommendations for people with type 2 diabetes, guidelines for dental follow-up, diabetic foot care, and the effects of stress and alcohol on blood glucose.

Following study completion, 16/21 (76%) of the SMS group participants provided feedback on their experience (Multimedia Appendix 9, Table S6). Of the 21 SMS group participants, 5 did not attend their scheduled 12-month follow-up clinic visit (or a makeup appointment for their missed clinic visit); therefore, these study participants did not provide feedback on the program. Those who did not provide feedback on the program were slightly younger (age 29.2 years, SD 6.1 years, vs 34.2 years, SD 5.4 years) and heavier (40.4 kg/m^2^, SD 11.3 kg/m^2^, vs 29.1 kg/m^2^, SD 5.7 kg/m^2^) with slightly lower baseline HbA_1c_ (6.8%, SD 0.9%, vs 7.4%, SD 1.8%).

All respondents to the end-of-study survey found that the study messages were easy to understand, supportive in nature, and delivered at appropriate times. In addition, 94% (15/16) of respondents reported that the messages contained practical information for people with diabetes and motivated them to think about their diabetes management. Overall, 94% (15/16) of respondents indicated that they would recommend the program to all new patients of the clinic.

## Discussion

### Principal Results

The TEXT2U study established that an enhanced SMS text message–based support and reminder program was effective at improving scheduled clinic attendance rates. Relative to the control group, the enhanced SMS text messaging intervention more than doubled the number of participants who attended all follow-up appointments. For those who attended all scheduled clinic appointments, the overall finding was stability of HbA_1c_, weight, and lipid profiles during the 12-month study period. With respect to the psychological impact of the intervention, we observed an improvement in psychosocial self-efficacy; a statistically significant favorable change in DES-SF score was observed at 6 months. However, we did not observe any between-group differences in measures of diabetes-specific distress nor in measures of perceived and experienced stigma related to living with type 2 diabetes. Overall, the enhanced SMS text message–based support and reminder program was well received and facilitated between-visit engagement with the health care team; >90% of respondents to the end-of-study survey said they would recommend the program to new patients. Although the completion rate of the end-of-study survey was 76% (5 participants did not attend a 12-month study visit), it remains reassuring that >70% of all SMS program recipients found utility in the SMS text messaging program employed in this study. In this setting, one anticipates that the majority of future consumers of TEXT2U will derive benefit from participation in the program.

### Research in Context

Our findings align with existing evidence that indicates text messaging interventions are an effective method to aid in the modification of health behaviors [11,13]. In a diabetes-specific context, the Sweet Talk RCT [26] identified that a text messaging system to support young people with type 1 diabetes was successful in improving measures of diabetes self-efficacy but not glycemic control. It is well recognized that the needs of youth with type 1 diabetes differ from those with type 2 diabetes [27]. Consequently, it is informative that this is the first study to show clear benefit of an enhanced SMS text message–based intervention over and above simple SMS reminder text messaging on clinic attendance in young-onset type 2 diabetes.

The issues of suboptimal clinic attendance and loss to follow-up are important problems in clinical practice. Maintaining clinic attendance is particularly important during young adulthood given the increased risk of diabetes complications [6] and the competing demands of family, work, study, and socializing, which interfere with maintenance of optimal care. Recurrent nonattendance prevents timely identification of changes in an individual’s health status and eliminates the opportunity for diabetes complications screening, early intervention, and ongoing education; moreover, it hinders the development of a therapeutic relationship [28]. Various strategies, ranging from written, verbal, and electronic reminders to expensive case management approaches, have been used by health service providers in an attempt to improve clinic attendance and patient outcomes [29,30]. This study adds to the literature by demonstrating that a low-cost, enhanced SMS text message–based intervention is effective at improving clinic attendance over and above a standard SMS appointment reminder system for those with young-onset type 2 diabetes, a cohort that is arguably at greatest risk for poor diabetes outcomes.

Nevertheless, maintenance of optimal clinic attendance remained a significant challenge, with just over one half of the enhanced SMS group and one-quarter of the control group recording a 100% attendance record. The factors that influence clinic nonattendance in youth with type 2 diabetes are poorly understood, likely to be complex, and an important area for further study.

In addition to adverse effects for the individual, nonattendance reduces clinic efficiency and places a significant burden on administrative staff, who are required to contact nonattenders and reschedule make-up appointments [31]. Notably, the enhanced SMS text messaging intervention resulted in improved clinic attendance rate without additional administrative burden. Overall clinic attendance rates were boosted to 80% in both groups; however, with additional administrative intervention to contact patients after missed appointments and then reschedule makeup appointments, this requirement was much higher for the control group. In a resource-constrained setting, it is highly likely that youth who miss clinic appointments will be lost to follow-up and therefore at increased risk of poor long-term outcomes. Moving forward, in-built automation of this program will allow the intervention to be scaled with minimal demand on clinic resources. Although a formal cost-benefit analysis was not undertaken, the relatively low cost of SMS text messaging ensures that this program is not prohibitively expensive.

It is well accepted that improving engagement of patients with their own health care can improve outcomes, and the concept of an informed and empowered patient is now central to many models of care [32-34]. The DES-SF measures diabetes self-efficacy across three main domains: (1) managing psychosocial aspects of diabetes care, (2) assessing dissatisfaction and readiness to change, and (3) setting and achieving goals [19]. For those who attended follow-up appointments in our study, we observed a more favorable pattern of response with respect to serial DES‑SF scores in the intervention group. Conceivably, delivery of supportive text messages and the opportunity to clarify queries by SMS text messaging between clinic visits in our study facilitated an increased sense of empowerment. Furthermore, incorporation of knowledge-based messages allowed important clinical information (including dietary and lifestyle recommendations) to be reinforced outside the clinic setting. This is particularly relevant given that postconsultation retention of medical information is poor [35].

The lack of a statistically significant improvement in DES-SF score at 12 months may indicate that the support provided by the enhanced SMS text messaging intervention has limited durability or that there may be a threshold to any improvement seen. Furthermore, we did not demonstrate improvement in measures of distress or stigma; plausibly, these more negative psychological elements respond best to in-person encounters and are not amenable to a text-based intervention. Although diabetes empowerment was supported by the enhanced SMS text message–based intervention, we did not see improvement in objective measures of self-care as evidenced by the availability of the SMBG/pathology results.

Although the intervention increased scheduled clinic attendance, we did not observe a difference in mean HbA_1c_, weight, or lipids between the intervention and control groups at study completion. For the most part, this is unsurprising given that the overall attendance (ie, scheduled attendance plus attendance at makeup appointments) was equal in both study arms. Irrespective of whether a participant attended a scheduled or makeup appointment, those who attended were afforded the same opportunity to have their management adjusted by the treating clinician. Nonetheless, stability of glycemia, close to target HbA_1c_, over 12 months of follow-up is not an insignificant achievement. The TODAY study clearly demonstrated that many adolescents with type 2 diabetes struggle to maintain glycemic control despite comprehensive care in a well-supervised setting [36].

### Study Strengths

The age of the study cohort and their familiarity with text messaging allowed for immediate engagement with the SMS text messaging program; individuals were not hampered by a learning curve in deriving benefit from study participation. In designing this study, we were mindful of the need to avoid overwhelming participants with too many messages; prevention of participant withdrawal due to message fatigue was an important consideration. In previous studies of SMS interventions, investigators regularly sent participants multiple text messages every week. The length of most previous studies has been comparatively short (usually <6 months), and participant attrition due to message fatigue in the short term is less likely. Given our 12-month study duration, a conscious decision was made to taper message frequency over the course of the study. Certainly, there is evidence to suggest that text messaging interventions with a tapering frequency have higher efficacy [37]. Personalization of the text messages ensured that the messages received by each participant were relevant to their personal circumstances, and the intervention had high acceptability. Feedback revealed that 88% of participants felt they had received an appropriate number of messages and 100% felt they received messages at appropriate times. Of the 21 enhanced SMS text messaging program participants, 20 opted to receive all study text messages; study message fatigue was not a significant issue.

### Limitations

There are several limitations of this study. First, it should be noted that our study was powered to detect a difference in clinic attendance but not to detect small differences in secondary outcomes. Secondly, our study was conducted at a secondary and tertiary referral center with a modest number of participants; testing in other settings will be required to confirm generalizability. In addition, our study population comprised a well-educated cohort (80% had attended, or were attending, Technical and Further Education [TAFE] or university). Although the message program employed plain language, it would be worthwhile to consider further testing in groups with lower literacy levels to establish better generalizability of the results. Furthermore, we specifically focused on the younger demographic; therefore, applicability in older age groups remains uncertain. Although all study participants had sufficient English proficiency, given our multicultural population, provision of the program in a participant’s native language may well have resulted in greater engagement. By design, it was not possible to blind both participants and treating clinicians in this study. However, blinding of treating clinicians and the use of an objectively measured primary endpoint were important aspects of the study design that help to mitigate observer bias.

### Conclusions

In young-onset type 2 diabetes, a low-cost, automated, enhanced SMS text message–based support and reminder system improved scheduled clinic attendance and diabetes self-efficacy over 12 months of follow-up. These achievements were made with high acceptability and low administrative burden. This text-based intervention has the potential to have substantial impact on the care of youth with type 2 diabetes at an especially vulnerable time of life.

## Appendix

Multimedia Appendix 1 Examples of text messages included in the TEXT2U study.

Multimedia Appendix 2 Enrollment and participant flow in the TEXT2U randomized controlled trial.

Multimedia Appendix 3 Overall attendance patterns for the intervention and control groups of the TEXT2U study.

Multimedia Appendix 4 Mean change in selected metabolic indices (95% CI) after 12 months for the intervention and control groups of the TEXT2U study.

Multimedia Appendix 5 Proportion of participants with SMBG data available at clinic study visits.

Multimedia Appendix 6 Proportion of participants with pathology data available at clinic study visits.

Multimedia Appendix 7 Changes in validated measures of psychosocial status throughout the study.

Multimedia Appendix 8 Overview of participant utilization of the optional SMS portal.

Multimedia Appendix 9 Utility and acceptability of the enhanced SMS program to the intervention participants.

Multimedia Appendix 10 CONSORT-eHEALTH checklist (V 1.6.1).

## Abbreviations

BP: blood pressure
DES-SF: Diabetes Empowerment Scale–Short Form
DSAS-2: Type 2 Diabetes Stigma Assessment Scale
HbA_1c_: hemoglobin A_1c_
OR: odds ratio
PAID-5: Problem Areas in Diabetes 5-item Short Form
RCT: randomized controlled trial
REDCap: Research Electronic Data Capture
RPAH: Royal Prince Alfred Hospital
SMBG: self-monitoring of blood glucose
TAFE: Technical and Further Education
TODAY: Treatment Options for Type 2 Diabetes in Adolescents and Youth
