# Supplementary material for: An Enhanced SMS Text Message–Based Support and Reminder Program for Young Adults With Type 2 Diabetes (TEXT2U): Randomized Controlled Trial
Source: J Med Internet Res. 2021 Oct 21;23(10):e27263. doi: 10.2196/27263 (PMC8569538; doi:10.2196/27263)
Supplement: Multimedia Appendix 1 [file jmir_v23i10e27263_app1.doc]

**Table S1:** Examples of text messages included in the TEXT2U study

**Informative**

*Having diabetes doesn’t mean you have to give up all your snacks. It’s often best to have smaller main meals with a snack between. Good snack options include half a dozen strawberries or a handful of your favorite nuts.*

**Supportive**

*Have you noticed sadness or worry getting in the way of your happiness? Talking about it with someone you trust often helps. Your diabetes care team is here for you, so let us know if you are feeling down.*

**Appointment Reminder**

*Hi Tim, just a reminder that your follow up appointment at the Diabetes Centre is booked at 3 pm Tuesday 26 June 2018. Please let us know if you are unable to attend so we can reschedule. Don't forget to upload your blood glucose meter and have your blood tests this week so we can review the results with you.*
