# Supplementary figures and images for: An Enhanced SMS Text Message–Based Support and Reminder Program for Young Adults With Type 2 Diabetes (TEXT2U): Randomized Controlled Trial
Source: J Med Internet Res. 2021 Oct 21;23(10):e27263. doi: 10.2196/27263 (PMC8569538; doi:10.2196/27263)

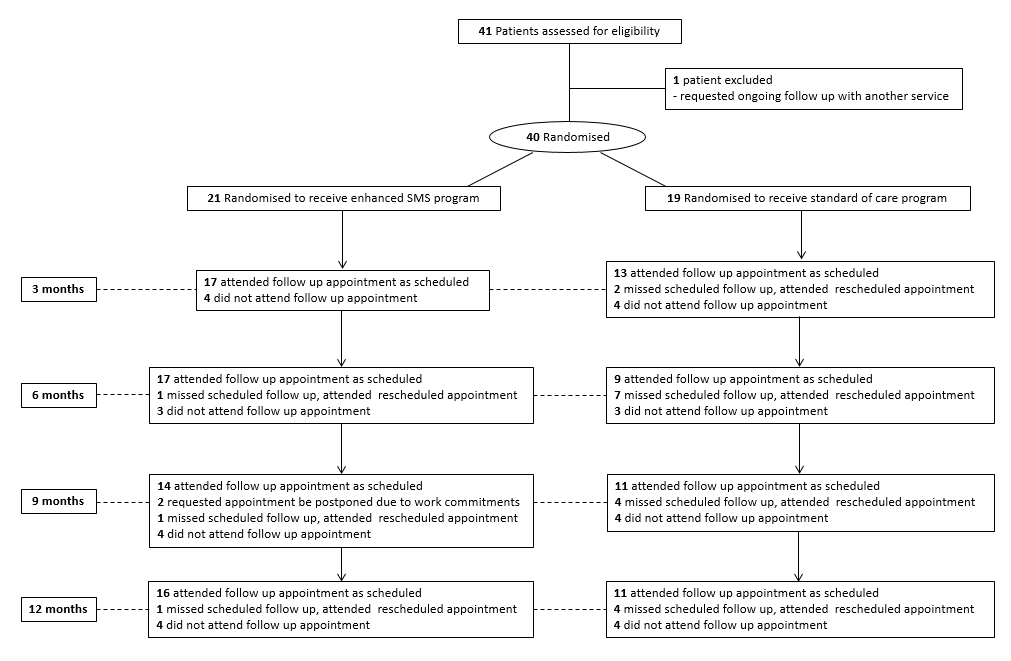


**Figure S1:** Enrolment and Participant Flow in the TEXT2U Randomized Controlled Trial

Supplement: Multimedia Appendix 2 [file jmir_v23i10e27263_app2.doc]

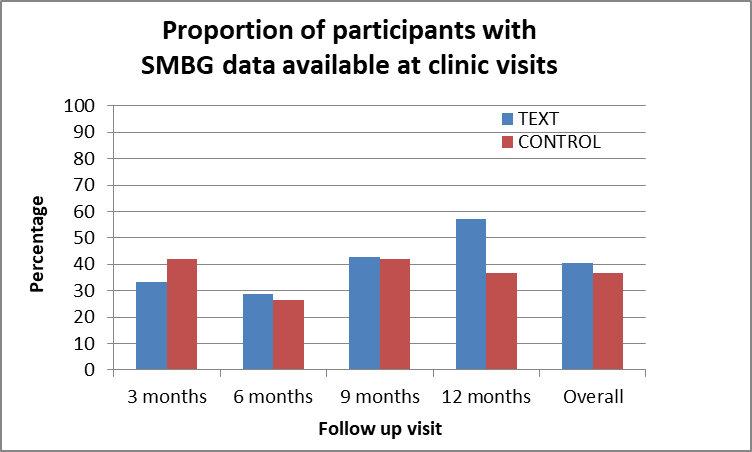


**Figure S2:** Proportion of participants with SMBG data available at clinic study visits

Supplement: Multimedia Appendix 5 [file jmir_v23i10e27263_app5.doc]

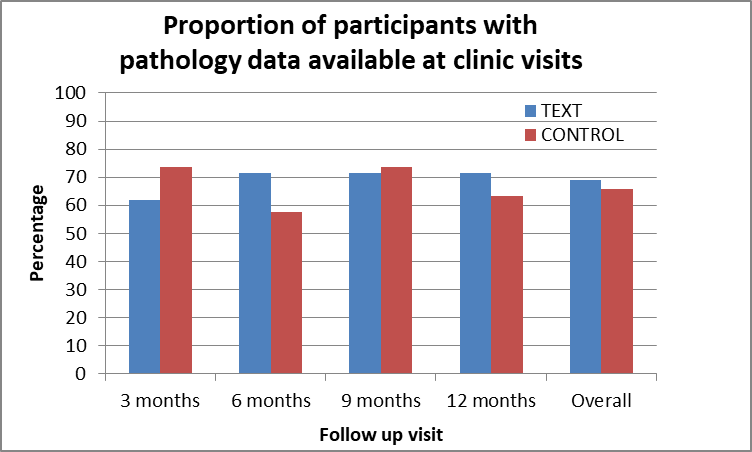


**Figure S3:** Proportion of participants with pathology data available at clinic study visits

Supplement: Multimedia Appendix 6 [file jmir_v23i10e27263_app6.doc]
