# Supplementary material for: An Enhanced SMS Text Message–Based Support and Reminder Program for Young Adults With Type 2 Diabetes (TEXT2U): Randomized Controlled Trial
Source: J Med Internet Res. 2021 Oct 21;23(10):e27263. doi: 10.2196/27263 (PMC8569538; doi:10.2196/27263)
Supplement: Multimedia Appendix 3 [file jmir_v23i10e27263_app3.doc]

**Table S2:** Overall attendance patterns for the intervention and control groups of the TEXT2U study

|  | **Intervention Group** | **Control Group** |
| --- | --- | --- |
| **Attended scheduled appointment n (%)** | 64 (76) | 44 (58) |
| **Attended make-up appointment n (%)** | 3 (4) | 17 (22) |
| **Did not attend either scheduled or make-up appointment n (%)** | 17 (20) | 15 (20) |
| **Total number of attendance outcomes n (%)** | 84 (100) | 76 (100) |
