# Supplementary material for: An Enhanced SMS Text Message–Based Support and Reminder Program for Young Adults With Type 2 Diabetes (TEXT2U): Randomized Controlled Trial
Source: J Med Internet Res. 2021 Oct 21;23(10):e27263. doi: 10.2196/27263 (PMC8569538; doi:10.2196/27263)
Supplement: Multimedia Appendix 4 [file jmir_v23i10e27263_app4.doc]

**Table S3:** Mean change in selected metabolic indices (95% CI) after 12 months for the Intervention and Control Groups of the TEXT2U Study

|  | **Intervention Group^a^** | ***P* value** | **Control Group^b^** | ***P* value** |
| --- | --- | --- | --- | --- |
| ∆HbA1c (%) | -0.2 (-0.9 to +0.6) | .63 | -0.3 (-1.8 to +1.1) | .64 |
| ∆BMI (kg/m^2^) | -0.1 (-0.9 to +0.7) | .76 | +0.1 (-1.5 to +1.7) | .89 |
| ∆Total Cholesterol (mmol/L) | -0.1 (-0.7 to +0.5) | .67 | +0.1 (-0.5 to +0.6) | .82 |
| ∆Triglycerides (mmol/L) | -0.6 (-1.3 to +0.1) | .09 | +0.8 (-1.4 to +2.9) | .45 |

1. Availability of Baseline and 12-month data for the Intervention Group:

20/21 data pairs available for HbA1c, Total Cholesterol and Triglycerides; 18/21 data pairs available for Weight

1. Availability of Baseline and 12-month data for the Control Group:

15/19 data pairs available for HbA1c and Weight; 14/19 data pairs available for Total Cholesterol and Triglycerides

**Note:** One-way ANCOVA did not demonstrate any significant differences between the intervention group and control group with respect to change in any of the metabolic indices (HbA1c, BMI, total cholesterol or triglyceride level) after controlling for baseline values (p>0.05 for all comparisons).
