# Supplementary material for: An Enhanced SMS Text Message–Based Support and Reminder Program for Young Adults With Type 2 Diabetes (TEXT2U): Randomized Controlled Trial
Source: J Med Internet Res. 2021 Oct 21;23(10):e27263. doi: 10.2196/27263 (PMC8569538; doi:10.2196/27263)
Supplement: Multimedia Appendix 7 [file jmir_v23i10e27263_app7.doc]

**Table S4:** Changes in Validated Measures of Psychosocial Status throughout the Study

|  | **TEXT Group** | **CONTROL Group** | ***P* value** |
| --- | --- | --- | --- |
| **Mean (±SD) Baseline PAID-5 Score**  **Mean (±SD) 6 month PAID-5 Score**  **Mean (±SD) 12 month PAID-5 Score**  **Proportion of participants recording a favorable**  **change in PAID-5 Score at 6 months (relative to BL)**  **Proportion of participants recording a favorable**  **change in PAID-5 Score at 12 months (relative to BL)** | 12.8 ± 4.0 [n=21]  10.4 ± 2.8 [n=19]  10.8 ± 3.3 [n=17]  13/19 (68%)  10/17 (59%) | 11.1 ± 4.4 [n=18]  11.1 ± 4.4 [n=16]  10.3 ± 4.4 [n=15]  8/16 (50%)  8/15 (53%) | .20  .60  .72  .27  .76 |
| **Mean (±SD) Baseline DES-SF Score**  **Mean (±SD) 6 month DES-SF Score**  **Mean (±SD) 12 month DES-SF Score**  **Proportion of participants recording a favorable**  **change in DES-SF Score at 6 months (relative to BL)**  **Proportion of participants recording a favorable**  **change in DES-SF Score at 12 months (relative to BL)** | 30.7 ± 3.8 [n=21]  32.8 ± 3.6 [n=19]  33.3 ± 4.2 [n=17]  13/19 (68%)  12/17 (71%) | 33.0 ± 4.4 [n=18]  31.9 ± 5.8 [n=16]  33.5 ± 4.9 [n=15]  5/16 (31%)  8/15 (53%) | .09  .58  .92  .03  .31 |
| **Mean (±SD) Baseline DSAS-2 Score**  **Mean (±SD) 6 month DSAS-2 Score**  **Mean (±SD) 12 month DSAS-2 Score**  **Proportion of participants recording a favorable**  **change in DSAS-2 Score at 6 months (relative to BL)**  **Proportion of participants recording a favorable**  **change in DSAS-2 Score at 12 months (relative to BL)** | 45.2 ± 14.6 [n=21]  44.3 ± 14.7 [n=19]  43.9 ± 12.7 [n=17]  10/19 (53%)  9/17 (53%) | 41.8 ± 13.3 [n=18]  40.9 ± 12.7 [n=15]  43.9 ± 13.1 [n=15]  8/15 (53%)  6/15 (40%) | .45  .49  .99  .97  .46 |
