# Supplementary material for: An Enhanced SMS Text Message–Based Support and Reminder Program for Young Adults With Type 2 Diabetes (TEXT2U): Randomized Controlled Trial
Source: J Med Internet Res. 2021 Oct 21;23(10):e27263. doi: 10.2196/27263 (PMC8569538; doi:10.2196/27263)
Supplement: Multimedia Appendix 8 [file jmir_v23i10e27263_app8.doc]

**Table S5:** Overview of participant utilization of the optional SMS portal

| Number (%) of the enhanced SMS group who sent at least one SMS to the team via the SMS portal | 11 (52) |
| --- | --- |
|  |  |
| Median (IQR) number of text messages sent to the team by participants in the enhanced SMS group | 3 (3-7) |
|  |  |
| Range of text messages sent to the team via the SMS portal | 1-29 |
|  |  |
| Thematic analysis of the content of messages sent to the study team via the SMS portal |  |
| - Expression of gratitude (for a message received or a service provided by the clinic) - Question (or clarification) regarding an aspect of diabetes management - Request to reschedule the timing of a follow-up appointment - Clarification (or confirmation) of the timing of a follow-up appointment / attendance at an appointment | 28%  25%  24%  8% |
