# Supplementary material for: An Enhanced SMS Text Message–Based Support and Reminder Program for Young Adults With Type 2 Diabetes (TEXT2U): Randomized Controlled Trial
Source: J Med Internet Res. 2021 Oct 21;23(10):e27263. doi: 10.2196/27263 (PMC8569538; doi:10.2196/27263)
Supplement: Multimedia Appendix 9 [file jmir_v23i10e27263_app9.doc]

**Table S6:** Utility and acceptability of the enhanced SMS program to the Intervention Participants^a^

| **Feedback Statement** | **Number (%) of Respondents**  **who Agree or Strongly Agree**  **with the Feedback Statement** |
| --- | --- |
| The text messages I received were easy to understand | 16 (100) |
| The messages included practical information for people with diabetes | 15 (94) |
| The text messages were supportive | 16 (100) |
| The text messages I received were relevant to me | 12 (75) |
| The text messages motivated me to think about my diabetes | 15 (94) |
| I liked being able to text in a question and then receive a text back from the Diabetes Centre with an answer | 13 (81) |
| Appointment reminder messages were helpful for me | 15 (94) |
| I received TEXT2U messages at appropriate times | 16 (100) |
| The number of messages I received during the study was appropriate | 14 (88) |
| I would recommend that the TEXT2U program be offered to all new patients of the young adult clinic | 15 (94) |

1. Response options to the Feedback Statements were “Strongly agree, Agree, Neither agree nor disagree, Disagree, Strongly Disagree”.

16 of the 21 (76%) participants enrolled in the enhanced SMS program completed a feedback form at the end of the study.
